# Intratumour heterogeneity in microRNAs expression regulates glioblastoma metabolism

**DOI:** 10.1038/s41598-021-95289-9

**Published:** 2021-08-05

**Authors:** Huda Alfardus, Maria de los Angeles Estevez-Cebrero, Jonathan Rowlinson, Amna Aboalmaaly, Anbarasu Lourdusamy, Salah Abdelrazig, Catherine Ortori, Richard Grundy, Dong-Hyun Kim, Alan McIntyre, Stuart Smith

**Affiliations:** 1 Children’s Brain Tumour Research Centre, School of Medicine, Faculty of Medicine and Health Sciences, University of Nottingham Medical School, Queen’s Medical Centre, Nottingham, NG7 2UH UK; 2 Division of Cancer and Stem Cells, School of Medicine, Faculty of Medicine and Health Sciences, University of Nottingham Medical School, Queen’s Medical Centre, Nottingham, NG7 2UH UK; 3 Centre for Analytical Bioscience, Advanced Materials and Healthcare Technologies Division, School of Pharmacy, The University of Nottingham, Nottingham, NG7 2RD UK

**Keywords:** Cancer, Molecular biology

## Abstract

While specific microRNA (miRNA) signatures have been identified in glioblastoma (GBM), the intratumour heterogeneity in miRNA expression has not yet been characterised. In this study, we reveal significant alterations in miRNA expression across three GBM tumour regions: the core, rim, and invasive margin. Our miRNA profiling analysis showed that miR-330-5p and miR-215-5p were upregulated in the invasive margin relative to the core and the rim regions, while miR-619-5p, miR-4440 and miR-4793-3p were downregulated. Functional analysis of newly identified miRNAs suggests their involvement in regulating lipid metabolic pathways. Subsequent liquid chromatography–mass spectrometry (LC–MS) and tandem mass spectroscopy (LC–MS/MS) profiling of the intracellular metabolome and the lipidome of GBM cells with dysregulated miRNA expression confirmed the alteration in the metabolite levels associated with lipid metabolism. The identification of regional miRNA expression signatures may underlie the metabolic heterogeneity within the GBM tumour and understanding this relationship may open new avenues for the GBM treatment.

## Introduction

Glioblastoma (GBM) is the most common type of adult brain tumour, representing 45.2% of all malignant brain and central nervous system tumours. GBM is the most aggressive (World Health Organisation (WHO) grade IV) and lethal form of glioma, an umbrella term for tumours thought to originate from glial progenitors. Even with the current treatment regimen which consists of maximal safe surgical resection followed by radiotherapy at 60 Gy with concurrent daily temozolomide (TMZ) followed by adjuvant TMZ, the prognosis of GBM patients remains poor with a median overall survival of 14 months and a 5-year survival rate of less than 10%, due to the high likelihood of tumour recurrence^1,2^. Glioma recurrence is often within 1–2 cm from the surgical resection margin appearing later on after the first diagnosis (metachronous) or being already present at the time of surgery but difficult to remove (synchronous)^3,4^. Yet, little is known about the molecular properties of the population of cells that remains behind after the surgical removal of the main mass of the GBM tumour.

The divergent development of subpopulations of cancer cells within the same tumour likely is one of the roots of therapy failure, the development of treatment resistance, and ultimately, recurrence of the malignancy^5^. A detailed understanding of how a tumour evolves through spatiotemporal pressures exerted via the locoregional space will provide an insight into the associated molecular mechanisms and allows the development of appropriate therapies. In GBM, intratumour heterogeneity in gene expression has been possible to study through the use of a fluorescence-guided multiple sampling (FGMS) approach based on 5-aminolevulinic acid (5-ALA) administration^6,7^. In this approach, viable tumour tissue can be identified by visible fluorescence and spatially distinct GBM tumour fragments can be defined during surgery. This method was used by Sottoriva et al.^9^ to perform a genome-wide transcriptional profiling of spatially distinct (superficial and deep) tumour fragments. Their work revealed that single tumours contain at least two different GBM subtypes (according to Verhaak classifier^8^). Moreover, they found that the most heterogeneously expressed genes within each tumour were involved in the same biological processes, such as brain cell proliferation/differentiation, neuronal activity, morphogenesis, angiogenesis and cell migration/invasion^9^.

MicroRNAs (miRNA) are a small non-coding RNAs of 20–25 nucleotides in length that regulate gene expression at post transcriptional level. miRNAs bind to complementary sequences within the 3′ untranslated regions (3′UTR) of gene transcripts and repress their translation into proteins. Over last 10 years, miRNAs have been shown to play a critical role in the GBM pathogenesis. Although changes in the expression levels of numerous miRNAs have been reported in GBM, spatial heterogeneity of miRNA expression within an individual tumour sample has not yet been investigated. Given that intratumour heterogeneity exists in GBM at the mRNA level, potentially orchestrated by miRNAs, this study aims to reveal the locoregional intratumour heterogeneity in miRNA expression, in a similar approach to studies conducted on mRNA expression, comparing spatially distinct regions of GBM tumours.

Here, we employ a fluorescence-guided multiple sampling (FGMS) approach based on 5-aminolevulinic acid (5-ALA) administration to isolate spatially distinct tumour fragments broadly categorised as core (central tumour), rim (brightly fluorescent more peripheral) and invasive (minimally fluorescent at resection limit) zones. This sampling technique enables us to collect tumour fragments (containing visibly fluorescent cells) from minimally fluorescent tissue at the resection margin, and crucially purify the neoplastic cells from the non-neoplastic parenchymal population. As such, we are able to collect a unique dataset from cells that are more likely to be representative of the cells remaining behind after surgery, and we also are able to interrogate intratumour miRNA expression heterogeneity across the entire tumour.

By comparing the miRNA expression profiles of spatially distinct regions of the same tumour, we show that there is a significant GBM intratumour heterogeneity in miRNA expression profiles. Moreover, we exemplify how, once a miRNA has been identified to be differentially expressed associated with specific region of the GBM tumour, miRNA target identification can link that region-specific information to the particular pathways influenced by those miRNAs. We do so by studying the functional role of two miRNAs associated with the invasive margin of GBM here through qRT-PCR, western blot, immunohistochemistry and liquid chromatography–mass spectrometry (LC–MS) and tandem mass spectroscopy (LC–MS/MS) analyses of GBM cells with dysregulated miRNA expression. Taken together, we demonstrate the involvement of those miRNAs in the regulation of lipid metabolic pathways in GBM.

## Results

### Intratumour heterogeneity of miRNA expression in primary tumours

To characterise miRNA intratumour variation, we performed microarrays to profile the miRNA expression (GeneChip miRNA 4.1 Arrays) in 45 tumour fragments from 15 adult GBM patients, two of whom were classified as *IDH* mutant (R132h), while the rest were classified as *IDH* wild-type (details of GBM cases are shown in Supplementary Table 1).

**Figure 1:**
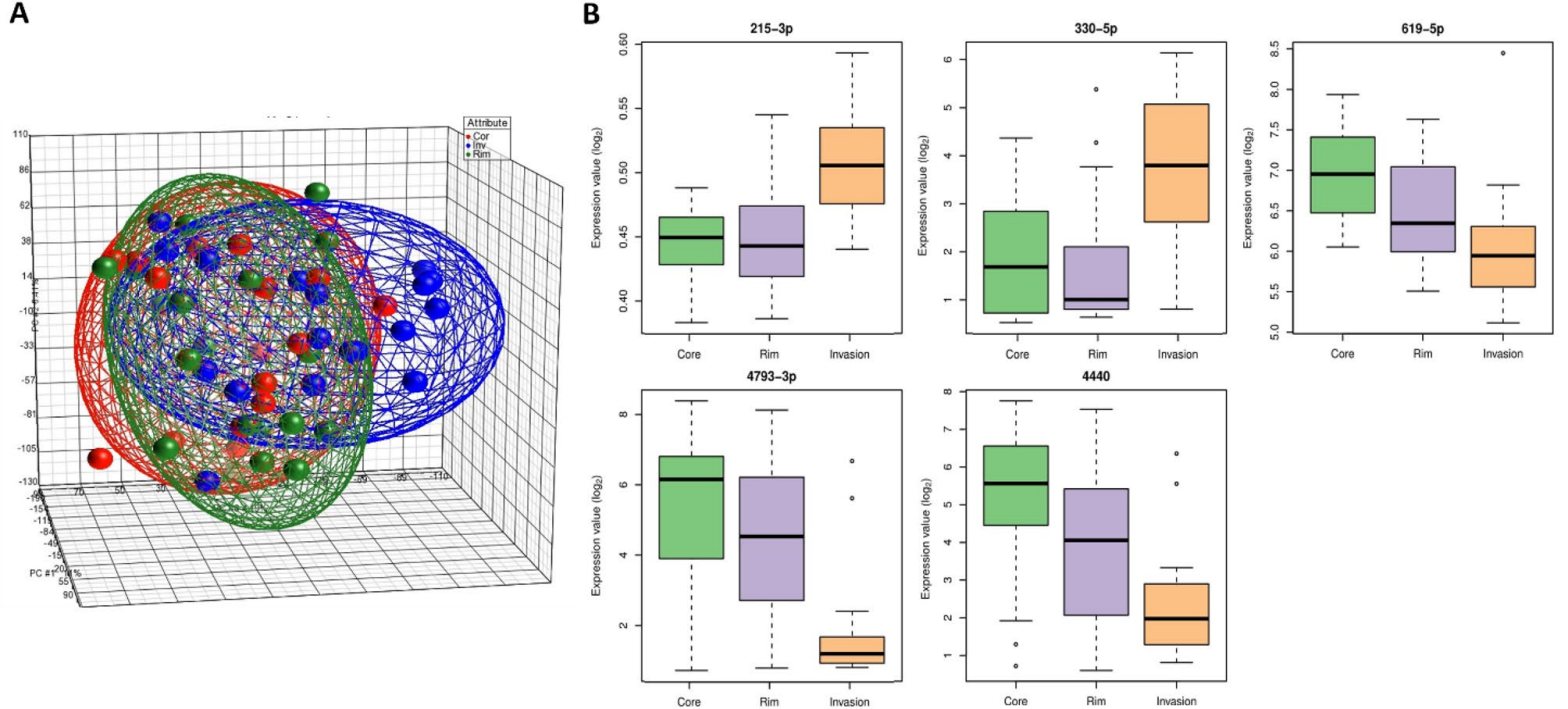
Intratumour heterogeneity in the expression profiles of miRNA in GBM. (**A**) Supervised analysis of miRNA expression profiles based on GBM tumour regions. PCA plot showing that the invasive margin region (blue) forms a cluster that is relatively distinct from the core (red) and the rim (green) regions; although all clusters overlapped due to the similarities between the different regions taken from the same patient tumour. (**B**) Statistically significant differentially expressed miRNAs identified by comparing different GBM tumour regions. miRNA expression values are shown for 15 patient tumours multi-sampled from the core (green), rim (purple), and invasive margin (inv; orange) regions. Statistical analysis was performed using moderated t-tests based on Bioconductor package limma. For the lines in a box and whisker plot: error bars are the 95% confidence interval, the bottom and top of the box are the 25th and 75th percentiles, the line inside the box is the 50th percentile (median), and any outliers are shown as open circles.

The similarities in the miRNA expression profiles between tumour regions were investigated by principal component analysis (PCA) (Fig. 1A). We found that the core and the rim had highly related miRNA expression profiles while the invasive margin was more distinct. Indeed, differential expression analysis comparing miRNA levels across the three GBM tumour regions in each patient sample revealed that two miRNAs were significantly upregulated in the invasive margin (miR-330-5p and miR-215-3p) while three miRNAs were significantly downregulated in the invasive margin (miR-619-5p, miR-4440, and miR-4793-3p) compared to the rim and the core regions (Table 1; Fig. 1B). Moreover, miR-330-5p, miR-4793-3p and miR-4440 showed relatively high fold changes in comparison between the invasive margin and the other two tumour regions, whereas miR-215-3p and miR-619-5p showed relatively lower fold changes. These results indicate that some miRNAs have varied expression levels within the different regions of the same tumour.

**Table 1:** Relative expression levels of differentially expressed miRNAs within the GBM tumour.

miRNA ID	Core vs rim			Rim vs invasive edge			Core vs invasive edge		
	Log(FC)	p-value	FDR	Log(FC)	p-value	FDR	Log(FC)	p-value	FDR
hsa-miR-215-3p	0.00	0.88	0.99	− 0.06	0.00	0.08	− 0.06	0.00	0.07
hsa-miR-4793-3p	0.66	0.39	0.99	2.65	0.00	0.35	3.32	0.00	0.07
hsa-miR-4440	0.99	0.15	0.99	1.68	0.02	0.56	2.67	0.00	0.18
hsa-miR-330-5p	0.19	0.70	0.99	− 2.04	0.00	0.10	− 1.85	0.00	0.23
hsa-miR-619-5p	0.46	0.07	0.99	0.46	0.07	0.68	0.92	0.00	0.23

miRNA expression for paired samples from 15 patients. Negative log2 transformed fold change (FC) values represent lower expression levels in the region written first.

*FDR* false discovery rate.

### qRT-PCR validation of the relative expression of the five differentially expressed miRNAs between core, rim and invasive margin regions

It is worth noting that using microarray technology for the analysis of a high number of variables at the relatively small number of samples can lead to false positive results even after applying stringent statistical analysis techniques^10^. As such, qRT-PCR was used to validate the microarray findings on an independent patient cohort. We performed qRT-PCR against the five differentially expressed miRNAs between core, rim and invasive margin regions (miR-330-5p, miR-215-3p, miR-619-5p, miR-4440, and miR-4793-3p). The *C. elegans* miRNA, cel-miR-54-3p, was used as an exogenous spike-in control^11^, while the hsa-miR-191-5p and hsa-miR-361-5p miRNAs were included as endogenous controls to generate normalised expression values. The endogenous controls were selected based on the literature^12^, the qRT-PCR manufacturers recommendations and by using the microarray data to identify miRNA which had the most relatively constant and moderately abundant expression across all tissue samples (See Supplementary Fig. 1).

**Figure 2:**
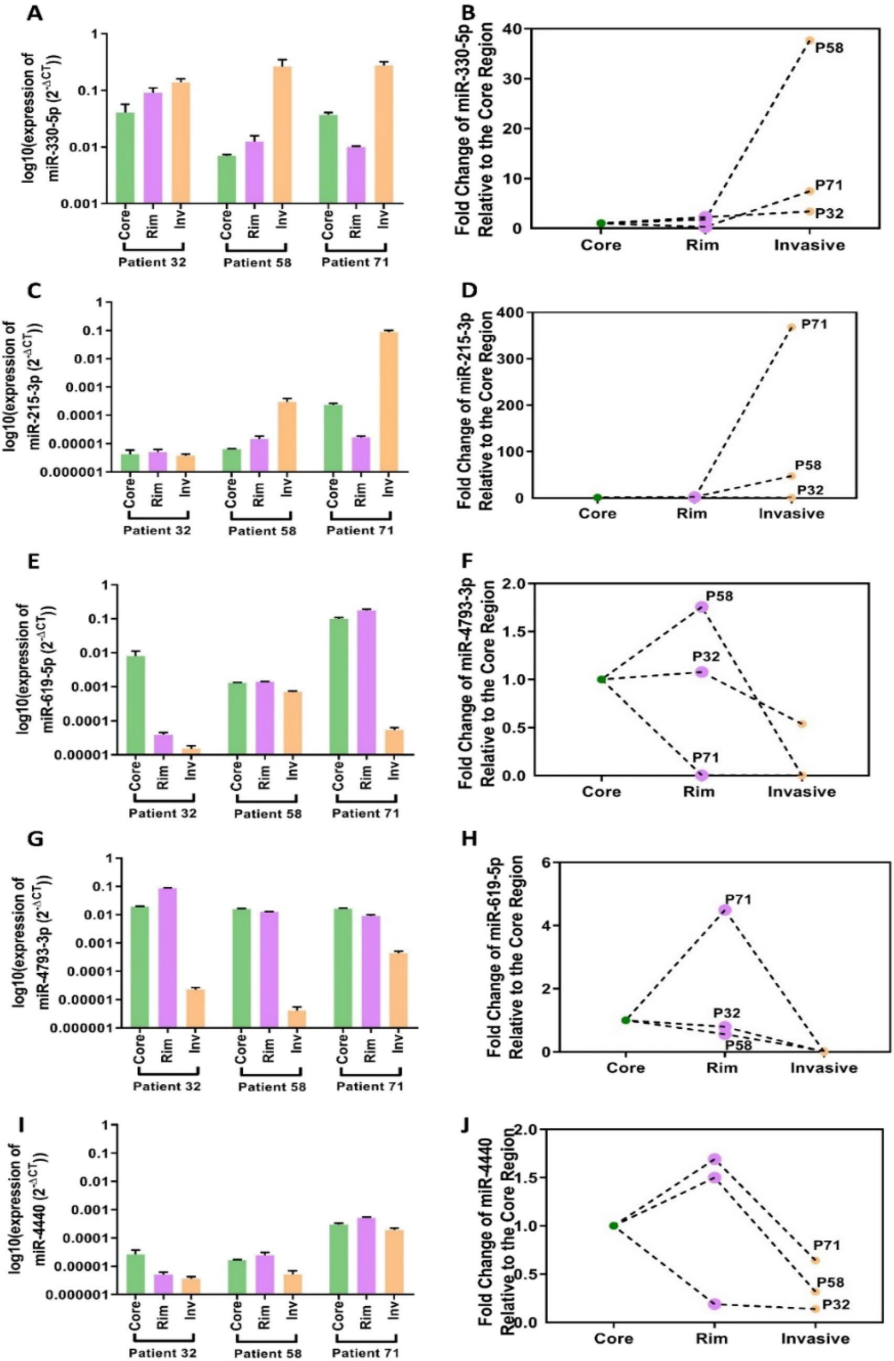
qRT-PCR validation of miRNA expression reported by microarray in an independent GBM patient cohort. Log of expression values for each tumour regions (left panel) and the fold change of the expression value in each tumour region relative to the core region (right panel) are shown for (**A**, **B**) miR-330-5p, (**C**, **D**) miR-215-3p, (**E**, **F**) miR-619-5p, (**G**, **H**) miR-4794-3p, and (**I**, **J**) miR-4440. Data is shown for individual GBM patients (n = 3). The numbers 32, 58, and 71 label the different patients. Error bars represent standard error of the mean (S.E.M) of three technical repeats. Statistical significance was not achieved due to the variation in the magnitude of the fold change in miRNA expression between each tumour region in different patients.

We found the qRT-PCR miRNA expression patterns to be consistent with those we had seen from the microarray (Fig. 2); with the exception of miR-215-3p that exhibited no differences between the tumour regions for one patient sample (GBM 32).

Thus, we were able to confirm that miR-330-5p and miR-215-5p were upregulated in the invasive margin relative to the core and rim regions, while miR-619-5p, miR-4440 and miR-4793-3p were downregulated.

### Differentially expressed miRNAs within the GBM tumour are predicted to target lipid metabolic pathways

**Figure 3:**
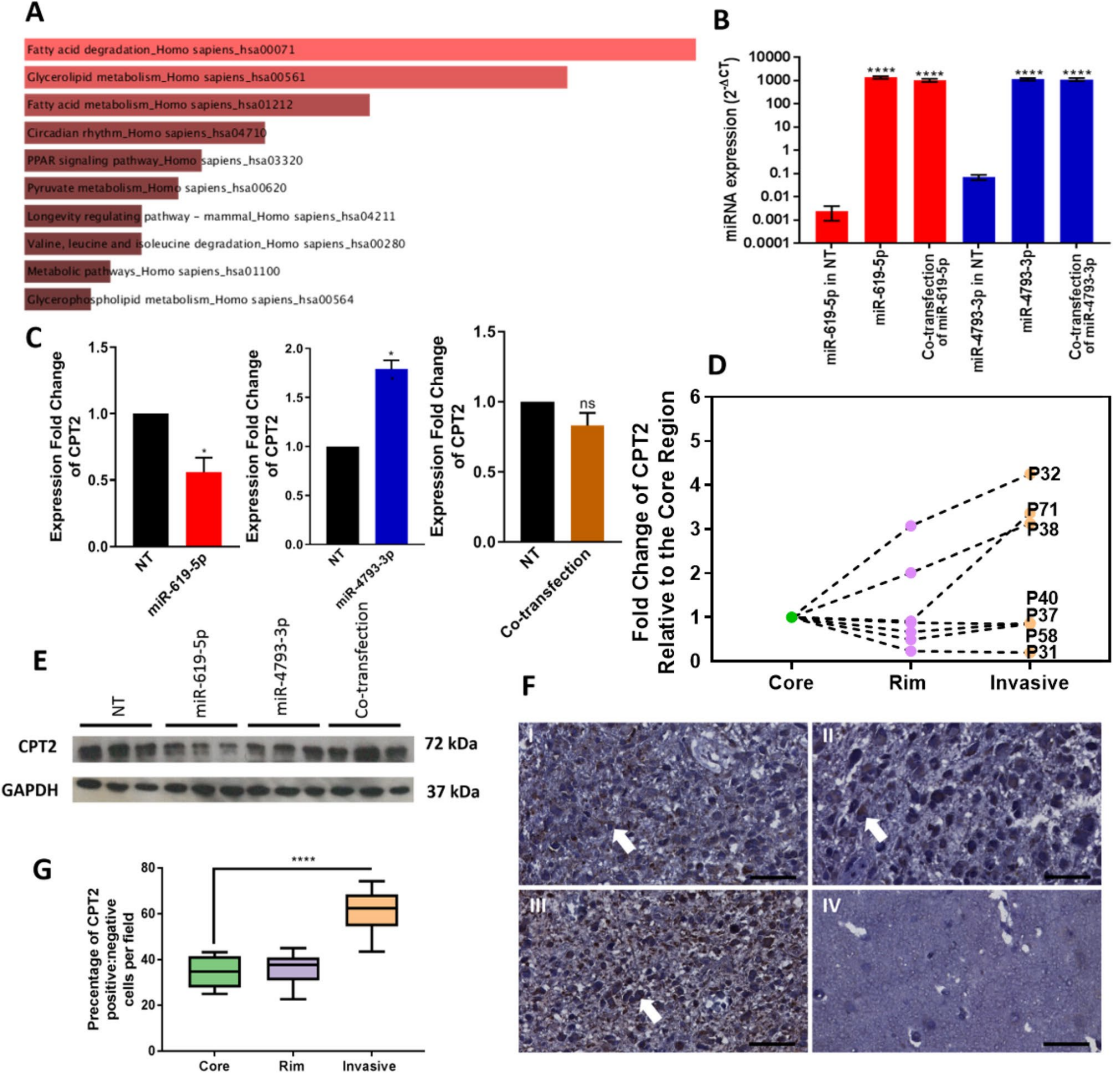
CPT2 as a putative target for miR-619-5p and miR-47933-3p. (**A**) Graphical representation of significantly enriched pathways for miR-619-5p and miR-4793-3p predicted gene targets. Colour shades from pink to black denotes declining levels of statistical significance. Significance was determined by two-sided sided z-score test (α < 0.05 following FDR correction). (**B**) Efficiency of miRNA knock-in transfection. qRT-PCR expression level (mean of three biological repeats ± S.E.M) of miR-619-5p (red bars) and miR-4793-3p (blue bars) in U87 cells after 24 h of transfection with either: non-targeting negative control (NT), miR-619-5p mimic (miR-619-5p), miR-4793-3p mimic (miR-4793-3p) or co-transfection with both miR-619-5p and miR-4793-3p at 1:1 ratio where 50 nM of each miRNA was simultaneously transfected. (****) p-value < 0.0001; one-way ANOVA followed by Bonferroni's multiple-comparison test. (**C**) CPT2 expression following miRNA overexpression. CPT2 expression values in U87 cells 48 h after the overexpression of miR-619-5p (red bar), miR-4793-3p (blue bar) or both miR-619-5p and miR-4793-3p at 1:1 ratio (brown bar) are represented as fold change (mean of three biological repeats ± S.E.M) relative to CPT2 expression in NT transfected cells. (*) p-value < 0.05; (ns) non-significant; two-sided t-test. (**D**) CPT2 expression in different regions of the GBM tumour. CPT2 expression is represented as fold change relative to its expression in the corresponding core region. Data is shown for individual patients (P32, P85, and P71). (**E**) Immunoblot of CPT2 in U87 cells. Full-length blots are included in the “Supplementary Information S1” file (Supplementary Fig. 3). All samples were loaded side-by-side on the same gel and the image of the blot was cropped around the bands that correspond to the protein of interest. CPT2 expression in U87 cells (n = 3) was evaluated after 72 h of transfection. GAPDH was used as a loading control. (**F**) Immunohistochemical staining of CPT2 in GBM tumours. Immunohistochemical staining of adult GBM tissue microarrays (TMA; n = 15) with anti-CPT2 antibodies (1:200 dilution) are shown as representative images of each region; tumour (I) core (II) rim (III) invasive margin and a (IV) normal brain control sample. Images were captured at ×40 magnification; scale bars correspond to 50 μm; arrows indicated positive staining. (**G**) The Number of cells containing CPT2-positive cytoplasmic staining represented as a percentage of the total number of cells counted on each high-powered microscope fields of view. Each region was compared to the core. (****) p-value < 0.0001; one-way ANOVA followed by Bonferroni's multiple-comparison test.

In order to shed light on the potential impact of the differential expression of miRNAs within the different regions of the same tumour, we interrogated the biological function of two miRNAs that have not yet been studied in GBM, miR-4793-3p and miR-619-5p both of which displayed differential fold changes between the invasive margin when compared to the core and rim regions of the tumour. We first predicted the putative gene targets for miR-619-5p or miR-4793-3p using miRWalk 2.0, which relies on multiple algorithms to predict the binding of the miRNA seed region to the miRNA recognition elements (mRE) within its target 3′UTR and so targets that were not predicted by at least two algorithms were eliminated. We only considered targets that contain mRE within their 3′UTRs; because miRNA binding sites within coding regions and 5′UTRs are less frequent and appear less effective than those in the 3′UTR^13–15^. Next, we short-listed the target genes that were mutually predicted for both miRNAs and overlapped with previously validated for either miR-619-5p or miR-4793-3p (156 genes). Pathway enrichment analysis was carried out on the short-list of target genes using KEGG2016 database in Enricher. We found that lipid metabolism was the most significantly enriched pathway (Fig. 3A).

Next, we investigated Carnitine Palmitoyltransferase (CPT2) as a potential target gene for miR-619-5p and/or miR-4793-3p. Our rationale for this was based on CPT2 being the most significant gene identified from the target analysis with no role reported in relation to GBM. We evaluated the expression of CPT2 by qRT-PCR and Western blot analyses after individual transfection or co-transfection (1:1 ratio) of miR-619-5p and miR-4793-3p mimics. As both miR-619-5p and miR-4793-3p are expressed at higher levels in the centre of the tumour compared to the periphery, we wanted to understand the potential synergic effect of the two miRNAs on GBM cells.

qRT-PCR was used to evaluate the efficiency of the miRNA mimic transient transfection for each of the miRNAs relative to a non-targeting control (NT). We found that the expression of miR-619-5p or miR-4793-3p was significantly increased after transfection with either mimic sequence in U87 cells (Fig. 3B). Subsequently, we found that the mRNA levels of *CPT2* were significantly reduced only upon the overexpression of miR-619-5p (Fig. 3C). Furthermore, we found the CPT2 protein levels to be markedly reduced upon the overexpression of miR-619-5p and the overexpression of miR-4793-3p in U87 cells compared to NT; although the co-overexpression of both miRNAs together did not result in the reduction of CPT2 protein levels (Fig. 3E).

Since the expression of both miR-619-5p and miR-4793-3p were found to be upregulated in the tumour core and rim relative to the invasive margin region, the expression of their putative target, *CPT2*, was expected to be downregulated in the tumour core and rim relative to the invasive margin. The mRNA levels of *CPT2* were investigated by qRT-PCR in the core, rim and invasive margin samples of seven adult GBM patients (*IDH* wild-type). In each patient sample, the expression of *CPT2* was generally highest in the invasive margin, followed by the rim and the core regions, except for patient 31 (Fig. 3D). We observed a large inter-patient variation in the magnitude of the fold change in gene expressions for each tumour region. Due to this, statistical significance was not achieved when comparing the average gene expression values for each tumour region across patients.

When conducting immunohistochemical staining of GBM tissue microarrays (TMAs), we found that CPT2 expression was higher in the invasive edge relative to the tumour core and rim regions (Fig. 3F). Subsequently, in each tumour region, we calculated the percentage of cells containing CPT2-positive cytoplasmic staining per field of view to determine statistical significance (Fig. 3G). Collectively, these results demonstrated that CPT2 might be targeted by miR-619-5p and/or miR-4793-3p in GBM.

### Differential expression of miRNAs leads to global metabolic changes in GBM cells

There is no study has been done to identify metabolites associated with miRNA expression changes in GBM cells, therefore, we employed LC–MS and LC–MS/MS profiling for the intracellular metabolome and lipidome of U87 cells 72 h after the overexpression of miR-619-5p, miR-4793-3p or both miRNAs at 1:1 ratio. Univariate analysis was performed to identify significantly changed metabolites following the deregulation of miRNA expression. The metabolite levels between NT and miR-619-5p overexpressing cells, between NT and miR-4793-3p overexpressing cells, and between NT and cells overexpressing both miR-69-5p and miR-4793-3p were compared. The levels of 40 metabolites were found significantly changed between the sample classes and due to the limitation of the database only 12 metabolites were identified (Table 2).

**Table 2:**
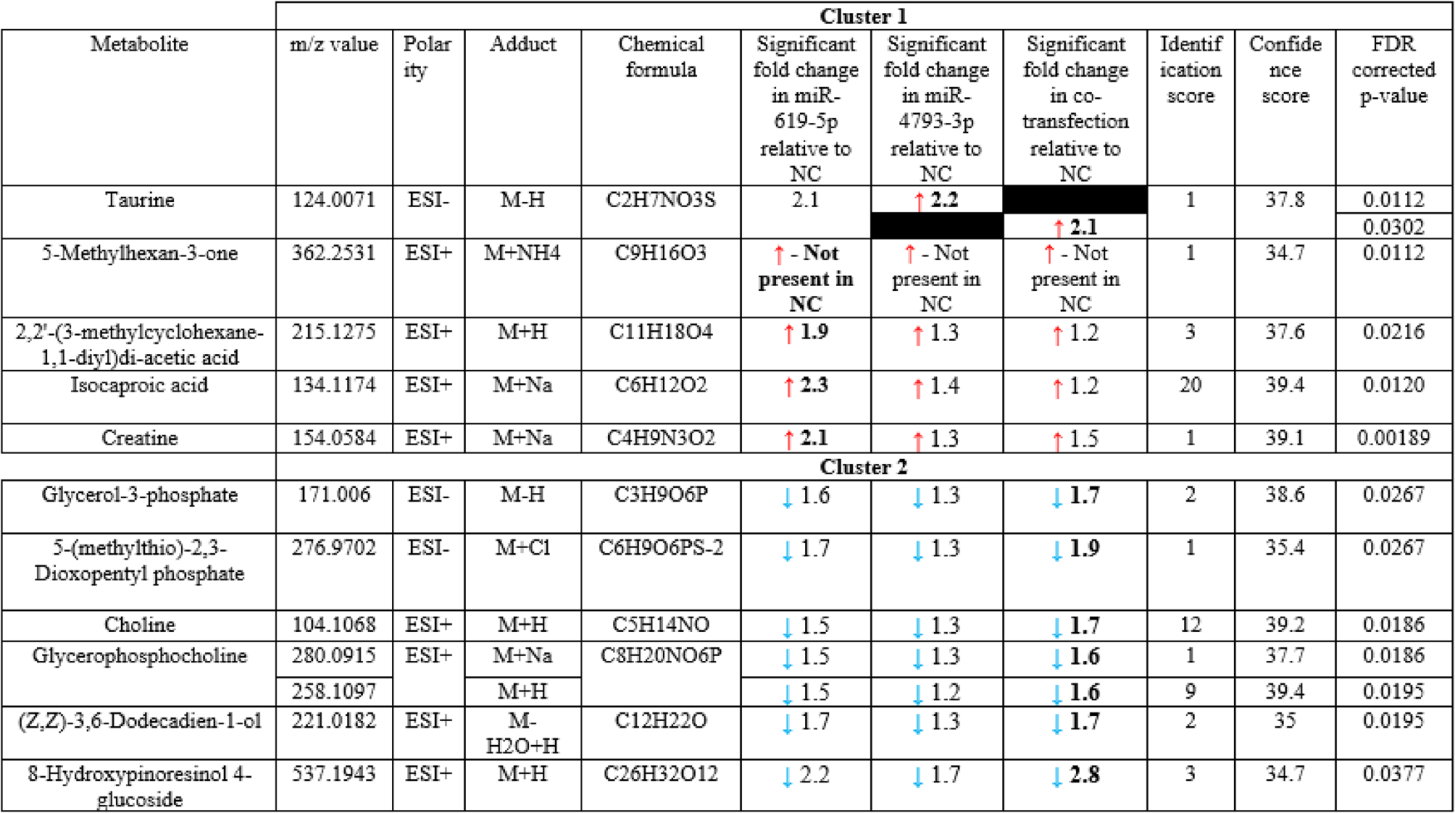
Fold change differences for identified differentially abundant polar metabolites.

Blue and red arrows represent significantly reduced or elevated metabolite levels relative to the non-targeting control (NT). Statistically significant fold changes are shown in bold. Statistical analysis was performed using a two-sided t-test, and p-values were corrected for multiple comparisons using the false discovery rate (FDR). Metabolites are identified either in the positive (ESI+) or the negative (ESI−) electrospray ionisation modes.

The abundance of 30 metabolites was found to be significantly altered between NT and co-transfection; while eight metabolites were found to be differentially abundant between NT and miR-619-5p and four metabolites showed significant alteration in their abundance between NT and miR-4793-3p.

**Figure 4:**
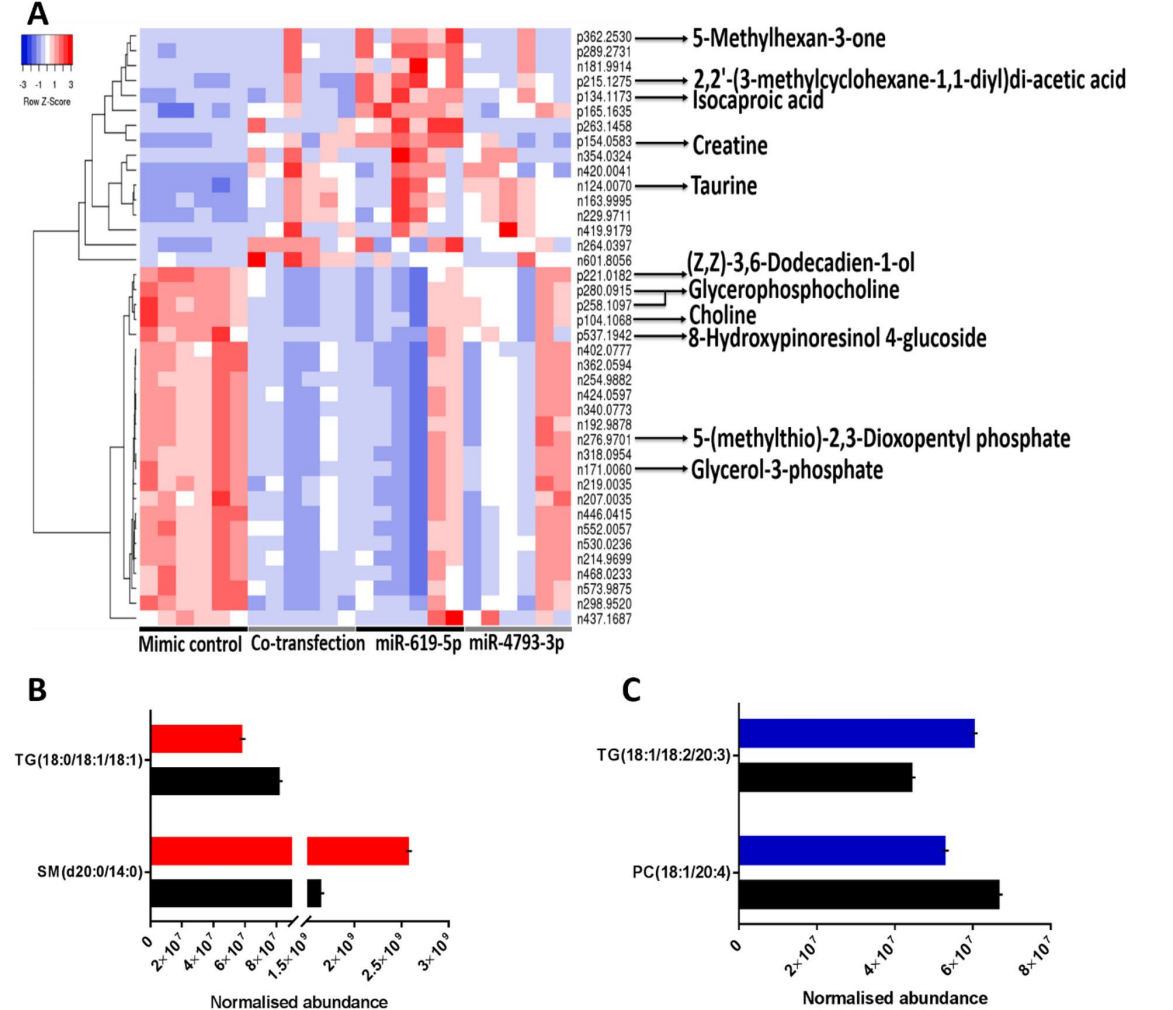
Global metabolic alterations following miR-619-5p and miR-4793-3p overexpression in GBM cells. (**A**) Hierarchical cluster analysis of differential metabolites in miR-619-5p, miR-4793-3p or miR-619-5p/ miR-4793-3p co-transfections relative to non-targeting control (NT). Shades of blue and red represent a decreased or increased abundance, respectively. Average linkage was applied as a clustering method, and the Spearman rank correlation was used as a distance measurement method. Abundance levels of significantly altered lipid species following (**B**) miR-619-5p and (**C**) miR-4793-3p transfections. Values are shown as mean of six biological repeats ± S.E.M. *PC* phosphatidylcholine, *SM* sphingomyelin, *TG* triglyceride.

A hierarchical cluster analysis of all differentially abundant metabolites is shown in Fig. 4A. Two significant hierarchical clustering groups were observed. Cluster 1 shows metabolites that were predominately decreased in NT compared to the other transfection conditions and cluster 2 shows metabolites that were increased in NT compared to co-transfection and miR-619-5p.

Five metabolites were identified from Cluster 1, and seven were identified from Cluster 2 (Fig. 4A). Such metabolites showed an average fold change of 2 between the transfection conditions. Each peak had a unique identification, except for glycerophosphocholine, which had two corresponding peaks in Cluster 2. Additionally, taurine was the only identifiable overlapping metabolite that contributed to the distinctions between NT and miR-4793-3p and between NT and miR-619-5p/miR-4793-3p co-transfections. We subsequently performed metabolic pathway enrichment analysis on those metabolites. Based on the analysis from MetaboAnalyst 3.0, the altered metabolites were mapped to nine key metabolic pathways (Table 3). Glycerophospholipid was the only significantly enriched pathway for choline, glycerophosphocholine and glycerol-3-phosphate.

**Table 3:** Metabolic pathway enriched for the significantly changed metabolites following miRNA transfection.

Pathway	Total	Hits	Names of metabolites	Raw p value	− Log(p)	FDR	Impact
Glycerophospholipid metabolism	39	3	Choline, glycerol-phosphocholine and glycerol-3-phosphate	7.6E − 05	9.483	0.006	0.110
Glycine, serine and threonine metabolism	48	2	Creatine and choline	5.6E − 03	5.194	0.222	0.034
Taurine and hypotaurine metabolism	20	1	Taurine	4.9E − 02	3.018	1.000	0.261
Ether lipid metabolism	23	1	Glycerol-phosphocholine	5.6E − 02	2.882	1.000	0.000
Glycerolipid metabolism	32	1	Glycerol-3-phosphate	7.7E − 02	2.561	1.000	0.044
Nitrogen metabolism	39	1	Taurine	9.3E − 02	2.370	1.000	0.019
Primary bile acid biosynthesis	47	1	Taurine	1.1E − 01	2.192	1.000	0.035
Cysteine and methionine metabolism	56	1	2,3-Diketo-5-methylthiopentyl-1-phosphate	1.3E − 01	2.026	1.000	0.025
Arginine and proline metabolism	77	1	Creatine	1.8E − 01	1.729	1.000	0.029

Fisher's exact test was applied for the enrichment analysis, and Degree Centrality was chosen for the topology analysis. p-values for each matched pathway was calculated from the pathway enrichment analysis (threshold was set to 0.05 following FDR correction), and the impact-value was calculated from the pathway topology analysis (threshold was set to 0.10). Hits refer to the number of metabolites identified in our study out of the total number of metabolites within a given pathway.

Lipidomics analysis was also performed to compare lipid profiles of the cells overexpressing miR-619-5p, miR-4793-3p or both miRNAs were compared to NT. While 6201 and 3577 lipid features were detected in positive and negative ionisation modes, respectively; a total of 382 lipid species were putatively identified using LipidSearch. Different levels of confidence in identification were assigned for the lipids based on class and fatty acid structure. Four lipids were identified as significantly altered between the different transfection conditions (Fig. 4B,C). The contents of the triglyceride species [TG (18:0/18:1/18:1)] were 1.4 fold lower in GBM cells overexpressing miR-619-5p compared to the control cells, while triglyceride species [TG (18:1/18:2/20:3)] were 1.3 folds higher in GBM cells overexpressing miR-4793-3p compared to the control cells transfected with NT. The phosphatidylcholine [PC (18:1/20:4)] was 1.4 folds lower in miR-4793-3p than in NT. Finally, the sphingomyelin [SM (d20:0/14:0)] exhibited 1.6 times higher levels in miR-619-5p relative to NT. No significant change in lipid species was observed when comparing NT and co-transfection.

## Discussion

There has been a focus on using samples obtained from the tumour core region of GBM as the subject of research to develop targeted therapies. However, such therapies have failed to improve patient survival in clinical trials. This disparity between pre-clinical and clinical results may be attributed to the heterogeneous molecular profile and clinical behaviour of the different regions of a GBM tumour^16^.

Although the approach of multi-region sampling has previously been employed to investigate genomic intratumour heterogeneity^9^, this is the first attempt to use this approach to determine the regional miRNA expression heterogeneity in GBM. The analysis of miRNA microarray data from 15 adult GBM patients identified five miRNAs that were differentially expressed in the tumour core/rim regions compared to the invasive margin region of the tumour. The relative expression of those miRNAs was then validated by qRT-PCR in three independent patient samples.

Because the invasive margin region is of particular clinical relevance and it has not yet been studied in the context of miRNAs, out of all the possible miRNA identified in previous in vitro studies, miRNAs identified as differentially expressed in the invasive margin region could serve as novel, and hopefully, more fruitful therapeutic targets.

An intrinsic limitation of our study is that pair-wise differential expression analysis was carried out on microarray data comparing the difference in the expression profiles of the core, rim and the invasive margin regions, without normalising to the expression levels of normal brain tissue. There are no publicly available datasets for normal human brain compatible with the most up-to-date version of the microarray we used, which has the 5p-strand/3p-strand feature annotations. This, however, is not a major disadvantage as it has previously been shown that different expression values and fold changes can be observed in miRNA profiles of GBM depending on which reference non-neoplastic brain control it has been compared to^17^. Moreover, the invasive margin samples contain non-neoplastic mixture of cell types, including reactive astrocytes, microglia and T-lymphocytes that are adjacent to the tumour cells. Yet, those cells do not represent the cells found in the normal brain, as they could have been somewhat transformed by the influence of the surrounding neoplastic cells^18^ and thus cannot serve as normal brain control. It is also important to highlight that caution must be taken when interpreting these results as the percentages of vital tumour cells vary between regions. Indeed, our previous work^19^ indicated that core and rim regions contain a median of greater than 90% tumour cells with more variability in the invasive zone whilst the dominant signal remains a neoplastic one^20^.

The two miRNAs (miR-619-5p and miR-4793-3p) we identified and validated in this study, as being differentially expressed between the core, rim and invasive margin regions, have not been previously recognised as having a role in GBM. The DNA sequence encoding miR-619-5p is located within the intragenic region of the slingshot homolog 1 (*SSH1*) gene^21^. High plasma levels of miR-619-5p serve as a possible biomarker for prostatic cancer growth and dissemination beyond the prostatic capsule^22,23^. The only validated target gene for miR-619-5p is the nuclear long non-coding RNA, metastasis-associated lung adenocarcinoma transcript 1 (*MALAT1*), which was studied in the context of colorectal carcinoma (CC)^24^. Several other studies showed that *MALAT1*, which is upregulated in CC, promotes its cell proliferation, motility and is associated with hypoxia, chemoresistance and poor prognosis^25–28^.

miR-4793-3p, on the other hand, is encoded by exon 26 of the cadherin EGF LAG seven-pass G-type receptor 3 (*CELSR3*) gene. CELSR3 is an adhesion G protein-coupled receptor (*GPCR*) that is differentially downregulated in mesenchymal GBM compared to other GBM subtypes, thereby presenting itself as a potential inhibitor of mesenchymal differentiation^29^. miR-4793-3p targets, however, are not well-documented in the literature, with only one study reporting Toll-like receptor 4 (*TLR4*) as a putative target of the upregulated miR-4793-3p in small bowel tissues of infants with necrotising enterocolitis (NEC)^30^.

We determined that miR-619-5p and miR-4793-3p target genes were enriched within the lipid metabolic pathways. We have shown that the overexpression of miR-619-5p in U87 cells (which endogenously have very low expression of miR-619-5p) downregulates the mRNA and protein expression of *CPT2*, one of the predicted putative target genes. In patient samples, we found that the mRNA expression of *CPT2* was downregulated where miR-619-5p was upregulated. This suggests that *CPT2* was upregulated in the tumour invasive margin, where miR-619-5p is downregulated, relative to the tumour core/rim, where miR-619-5p miRNAs is upregulated. Yet, when we evaluated the expression of CPT2 protein in patients TMAs; however, due to the lack of a GBM-specific marker to co-stain with, it was not possible to conclude whether the observed CPT2 protein expression in the invasive margin is exclusively localised to the tumour cell population. As such, we could not make assertions regarding CPT2 protein expression in the infiltrating tumour cells in the invasive margin region compared to the tumour cells in the core/rim regions.

The role of miR-619-5p and miR-4793-3p in regulating the metabolites associated with the lipid metabolic pathways were also investigated. The overexpression of miR-619-5p and miR-4793-3p in U87 cells were found to downregulate the metabolites associated with, amongst other pathways, lipid metabolic pathways; which confirmed our findings from the miRNA target gene prediction and pathway enrichment analyses. Such findings suggest that the intratumour miRNA expression heterogeneity may give rise to intratumour metabolic heterogeneity in GBM. There has been only one study exploring the regional heterogeneity in GBM metabolism in which the intratumour heterogeneity in glucose metabolism in GBM was demonstrated by Santandreu et al. They used six GBM samples surgically removed in one block, then freshly sectioned, to isolate central and peripheral regions^31^. They revealed that isolated mitochondria from peripheral regions showed higher oxidative activity than those isolated from the central regions^31^. Since the mitochondrial abundance (measured by mitochondria DNA content) was invariant between the two regions, this finding was explained as a regional adaptation to the hypoxic gradient^31^.

In our study, we found lipid metabolic pathways to be downregulated upon the overexpression of miR-4793-3p and/or miR-619-5p. For instance, phosphatidylcholine is a major glycerophospholipid component of the phospholipid monolayer of the cell membrane, mitochondrial membrane and lipid droplets. The levels of phosphatidylcholine were found to be downregulated in U87 cells overexpressing miR-4793-3p compared with NT. Upstream components of the phosphatidylcholine biosynthesis pathway were also found to be downregulated. The CDP-choline pathway produces the majority of phosphatidylcholine in the cell^32^; and we found that choline levels were reduced after the miR-619-5p and miR-4793-3p individual or co-transfections. Several studies showed that phosphatidylcholine biosynthesis is closely linked to the availability of choline^33^. A study in human leukemic monocyte-like U937 cells showed that Lidocaine, a local anaesthetic, inhibited the uptake of choline into the cells and reduced phosphatidylcholine levels by limiting the availability of choline for the CDP-choline biosynthetic pathway^34^.

In the final step of the CDP-choline pathway, the choline head group is added onto a diacylglycerol (DAG) backbone to form phosphatidylcholine. DAG is made from glycerol 3-phosphate (G3P). We found the DAG precursor, G3P, to be significantly downregulated in cells overexpressing either miR-619-5p or miR-4793-3p or in cells overexpressing both miRNAs together. As such, the low availability of G3P could affect the levels of phosphatidylcholine.

Both G3P and choline are products of glycerophosphocholine (GPC) degradation by glycerophosphodiesterase (GDE); whereas GPC itself is a product of phosphatidylcholine degradation. We found that the levels of GPC were also decreased following miR-619-5p and miR-4793-3p individual or co-transfections.

Since the mitochondrial membrane is composed of phosphatidylcholine, choline deficiency has been associated with mitochondrial dysfunction, the loss of membrane potential and the generation of high levels of ROS in hepatocytes^35,36^. Interestingly, there was a significant upregulation of taurine, well-known neuroprotective antioxidant, in miR-4793-3p and co-transfection conditions (a similar pattern was observed in miR-619-5p). Furthermore, 5-(methylthio)-2,3-Dioxopentyl phosphate which is used for the synthesis of methionine, a precursor for taurine, is downregulated in cells transfected with miR-619-5p and miR-4793-3p; thereby suggesting the possibility that 5-(methylthio)-2,3-Dioxopentyl phosphate could have been used up in the cell in order to make more taurine.

Sphingomyelin (SM), another class of phospholipid that are abundant in cell membranes^37^, is made from phosphatidylcholine. We found that upon overexpressing miR-619-5p, while the levels of the phosphatidylcholine were decreased, the levels of SM were significantly increased compared to NT; likely reflecting changes to membrane structure due to aberrant biosynthetic reactions.

In conclusion, the intratumour heterogeneity in GBM gene expression profiles has recently been elucidated and as far as we are aware, this is the first study reporting profiles of miRNA expression in different regions of the GBM tumour. Designing effective therapy to prevent GBM cells from infiltrating further into the normal brain will require a detailed understanding of the regulatory process important to the cells that remain behind after surgery. Thus, it is hoped that our work can act as a step towards understanding how the differential expression of miRNA within the different regions of GBM can have an impact on the regulation of lipid metabolic pathways. This finding is particularly relevant to elucidate the molecular properties of those cells within the invasive margin of the tumour that may more closely resemble the residual cell population responsible for tumour recurrence.

## Methods

All methods were performed in accordance with the relevant guidelines and regulations.

### Patient tissue samples

Patient tissue samples were collected at Queens’s Medical Centre, Nottingham University Hospitals NHS Trust, Nottingham, UK, between 2013 and 2016 and written informed consent was obtained from all patients prior to surgery in accordance with The Human Tissue Authority Codes of Practice for Research. Independent neuro-pathological review confirmed GBM diagnosis for each case. Ethics approval for the use of tissue samples in this project was obtained from the National Research Ethics Service of the East Midlands Research Ethics Committee (Reference 11/EM/0076).

During the surgery, three tissue fragments were obtained from the same tumour separated by at least 1 cm. The fragments were labelled as core, proliferative rim and invasive margin regions (according to their position relative to the centre of the tumour; Supplementary Fig. 2).

### RNA isolation and miRNA microarray

Total RNA was isolated from 20 to 30 mg of tissue samples using Ambion mirVana miRNA isolation kit (Life Technologies) according to the manufacturer’s protocol. The quality of RNA was assessed on the Agilent 2100 bioanalyser (Agilent) using reagents from the Agilent RNA 6000 Nano Kit according to manufacturer’s instructions. Total RNA samples were analysed on GeneChip miRNA 4.1 Array (Affymetrix). The miRNA expression datasets were filtered into human mature miRNA and pre-miRNAs (total n = 4603). Principal Component Analysis (PCA) was carried out as a supervised clustering technique in Partek Genomics Suite software (version 6.6; Copyright 2016; Partek Inc.).The differential expression was determined using the Linear Models for Microarray Data (limma) package^38,39^ as part of the Bioconductor project^40^ in R-Studio (version 0.99.902; RStudio, Inc. Boston, MA, USA).

### Target prediction and pathway enrichment analysis

miRNA target genes were predicted by miRWalk 2.0^41^ (using TargetScan (Release 7.1), DIANA-microT-CDS (Version 5.0), mirDB, and mirMap with a prediction score cut-off of < − 0.2, > 0.7, > 60, and > 90). Mutual targets were integrated using the V-lookup function in Microsoft Excel (Microsoft Office version 2013). Pathway analysis of predicted miRNA target genes was carried out using KEGG2016 database in Enricher^42^.

### qRT-PCR analysis of miRNAs expression

Reverse transcription (RT) of miRNA templates was performed using TaqMan Advance miRNA cDNA synthesis kit (Thermofisher Scientific) according to manufacturer’s protocol with an exogenous spike-in control, cel-miR-54-3p from *C. elegans* added to the total RNA sample at the start of the RT step.

The miRNA relative expression was determined by qRT-PCR using the TaqMan Advanced miRNA qRT-PCR Assay kit (Thermofisher Scientific) according to manufacturer’s protocol in a CFX-96 Real-Time System with a C100 Thermocycler (Bio-Rad) using TaqMan Advanced miRNA Assay Probes for (hsa-miR-619-5p; 479103_mir), (hsa-miR-4793-3p; 480053_mir), (hsa-miR-330-5p; 478830_mir), (hsa-miR-215-3p; 478769_mir), (hsa-miR-4440; 479809_mir), (hsa-miR-191-5p; 477952_mir), (hsa-miR-361-5p; 478056_mir).

Normalised miRNA expression (R) values were calculated relative to the average expression values of two endogenous controls (hsa-miR-361-5p and hsa-miR-191-5p) and the spike-in control as shown in the equation below and as previously described in^32^. Statistical analysis was performed in GraphPad Prism 8.

$$R = \frac{{2^{{ - \left[ {\left( {Ct \left( {miR\, of\, interest} \right) - Ct \left( {Spike\, - in\, control} \right)} \right]} \right.}} }}{{2^{{ - \left[ {\left( {Ct \left( {average\, of\, endogenous \,controls} \right) - Ct \left( {Spike \,- in\, control} \right)} \right]} \right.}} }}$$

### qRT-PCR analysis of gene expression in tissue samples

Gene expression analysis from tissue samples was performed by qRT-PCR using the TaqMan RNA-to-CT 1-Step Kit (Thermofisher Scientific) and the TaqMan Gene Expression Assay Probes for CPT2 (ID Hs01092524_m1) and the endogenous control, β-actin (ID: Hs01060665_g1), in a CFX-96 Real-Time System with a C100 Thermocycler following manufacturer’s protocol. Relative gene expression was calculated using the Pfaffl equation^43^.

### Cell culture and miRNA transfection

U87 cells were obtained from the American Type Culture Collection (ATCC) and cultured in Dulbecco’s Modified Eagle Medium (DMEM; Gibco) supplemented with 10% foetal bovine serum (HyClone), 1% l-glutamine (Sigma-Aldrich) and 1% Penicillin/Streptomycin (Sigma-Aldrich).

For miRNA transfection, U87 cells were seeded in 6-well plates until 50–60% confluent. They were transfected using DharmaFECT 1 Transfection Reagent (at 1:100 dilution; Horizon Discovery) with miRNA mimic (50 nM) for miR-619-5p (Product ID: C-303062-00-0005), miR-4793-3p (Product ID: C-302420-00-0005) or Non-targeting Mimic Control (Cel-miR-67; Product ID: CN-001000-01-05). Co-transfection was performed using 50 nM of each miRNA mimic.

### qRT-PCR analysis of gene expression in cell lines

After 48 h of transfection, U87 cells were harvested and RNA was isolated using the Ambion mirVana miRNA isolation kit. RNA was treated with DNase I kit (ThermoFisher Scientific). cDNA was synthesised by preparing, per reaction, 1 µg of DNase-treated RNA with 100 pmol of Oligo-dT, 2 µl of 10 mM dNTP, 20 U RiboLock RNase Inhibitor and 200 U RevertAid RT in enzyme buffer (all reagents from ThermoFisher Scientific). The mix was incubated at 42 °C for 1 h then at 70 °C for 10 min.

The qRT-PCR reactions were carried out by mixing 10–20 ng cDNA with appropriate amounts of iQ SYBR Green Supermix (Bio-Rad) and primer mix in a total volume of 25 µl. Primer optimisation was carried out to test different ratios of forward and reverse primers and a standard curve was ran to determine the optimal forward and reverse primer ratio where no primer dimers products are seen in amplification and melting curves. CPT2 (Forward Primer: AGGCTGTGGGCTCCATCGCCTA, Reverse Primer: AGTCAGTCCAGTACATGAAGCCA); β-actin (Forward Primer: ATTGGCAATGAGCGGTTC, Reverse Primer: GGATGCCACAGGACTCCA). The reaction took place in a CFX-96 Real-Time System with a C100 Thermocycler. Relative gene expression was calculated using the Pfaffl equation.

### Western blotting analysis

After 72 h of transfection, proteins were extracted from U87 cells with cOmplete, Mini, EDTA-free tablets (Roche Diagnostics) and protein concentrations were assessed by Bio-Rad Protein Assay according to the manufacturer’s protocols. The protein samples (20 µg per sample) were isolated by sodium dodecyl sulfate polyacrylamide gel electrophoresis (SDSPAGE) and electroblotted onto Amersham Hybond P membrane (GE Healthcare). The membrane was blocked by 5% semi-skimmed milk for 1 h at room temperature with monoclonal antibodies for CPT2 (ab52818; Abcam) at 1:1000 dilution or GAPDH (ab8245; Abcam) at 1:1000 dilution. This was followed by incubation with a horseradish peroxidase-linked (HRP)-conjugated secondary anti-rabbit (7074S; Cell Signalling Technology) or anti-mouse antibody (97166S; Cell Signalling Technology) diluted at 1:2000. Antibody binding was assessed through chemiluminescent measurement using ECL Western Blotting Detection Reagents (GE Healthcare).

### Immunohistochemistry

Freshly collected tumour tissues were fixed with 4% paraformaldehyde, dehydrated in a graded ethanol series, cleared in a xylene solution and embedded in paraffin wax. Formalin-fixed paraffin-embedded (FFPE) blocks were made for each individual region. Cores of 0.6 mm in diameter were taken in triplicates from each FFPE sample and built into an empty a tissue microarray (TMA).

TMA sections of 4 µm were deparaffinised in xylene and rehydrated in 100% and 95% ethanol before heat-induced antigen retrieval in sodium citrate buffer (pH 6.0) in a pressure cooker (full pressure, 121 °C). Sections were washed in PBS, blocked with 20% normal goat serum (ThermoFisher Scientific) for 20 min at room temperature, and incubated with a primary rabbit polyclonal anti-CPT2 antibody diluted 1:200 (ab30532; Abcam) overnight at 4 °C. The sections were developed with Dako EnVision kit.

### Untargeted LC–MS and MS/MS analysis in cell lines

Bi-phasic liquid–liquid extraction (3:1:1 chloroform:methanol:water) was used for the separation and extraction of the metabolites (polar fraction, the aqueous layer) and the lipids (non-polar fraction, the organic layer) from the U87 cells after 72 h of transfection for LC–MS and MS/MS analyses. Briefly, the cell pellets were harvested in 400 µl of methanol, shaken at 4 °C for 30 min before 1200 µl of chloroform were added followed by 400 µl of water. The mixtures were then incubated for 1 h and centrifuged at 14,000×*g* for 10 min at 4 °C. The two immiscible layers were separated and dried under vacuum. The aqueous residues were reconstituted in 70 μl of methanol for metabolomics analysis, while the organic restudies were reconstituted in 100 μl of isopropanol for lipidomics analysis. metabolites).

In metabolomics analysis, LC–MS was performed on an Accela UHPLC system coupled to Exactive Orbitrap mass spectrometer (Thermo Fisher Scientific) as previously described^44^. A ZIC-*p*HILIC column (4.6 × 150 mm, 5 μm particle size; Merck Millipore) was used for metabolite separation and the column temperature was maintained at 45 °C. The mobile phases consisted of (A) 20 mM ammonium carbonate in water and (B) 100% acetonitrile. Linear LC gradient was performed from 80% (B) to 5% (B) over 15 min then the compositions of A and B were returned to their initial conditions in 2 min and the column was left to re-equilibrate for 7 min (24 min total run time). The mass spectrometer was operated in simultaneous positive and negative ionisation modes. In positive ion mode, spray voltage was 4.5 kV and the capillary voltage was 20 V, while in negative ion mode, spray voltage was − 3.5 kV and capillary voltage was − 15 V. Sheath, auxiliary and sweep gas flow rates were 40, 5 and 1 (arbitrary unit), respectively, for both modes. Capillary and heater temperatures were maintained at 275 °C and 150 °C, respectively. Data were acquired in full scan mode with a resolution of 50,000 and a mass range of *m/z* 70–1400 with 4 Hz scan rate.

For lipidomics analysis, LC–MS/MS was performed on a Dionex Ultimate 3000 UHPLC System coupled to a Q-Exactive Orbitrap mass spectrometer (Thermo Fisher Scientific). A reverse-phase ACE Excel 2 C18 column (50 × 2.1 mm; 2 µm particle size; ACE) was used for the separation of lipids and the column temperature was maintained at 50 °C. The mobile phase consisted of (A) 60% or (B) 10% of 0.1% MS-grade ammonium acetate in 18.2 MΩ water with 40% (in the case of A) or 10% (in the case of B) of acetonitrile and 80% isopropanol (in the case of B). Both mobile phases were modified with 0.1% ammonium acetate abnd the gradient adopted was as follows: at 0 min, B was at 30% with a flow rate of 0.4 ml/min; at 1 min, B was at 35% with a flow rate of 0.4 ml/min; at 7.5–10 min, B was at 100% with a flow rate of 0.4 ml/min; at 11 min, B was at 100% with a flow rate of 0.5 ml/min; and finally, at 12–15 min, B was at 20% with a flow rate of 0.5 ml/min.

MS analysis was performed in a full scan mode (*m/z* 150–1500 with a resolution of 70,000) using simultaneous positive and negative ESI modes. The spray voltage was set to 3.5 (ESI+)/− 3.5 kV (ESI-) and flow rates of sheath gas, desolvation gas and sweep gas were 47.5, 11.25 and 2.25 units, respectively. The capillary and desolvation heater temperatures were set at 225 °C and 400 °C, respectively. The automatic gain control (AGC) target for MS acquisitions was set at 3 × 10^6^ ions with a maximum ion injection time of 100 ms. Tandem data-dependant MS/MS spectra were obtained on the most intense 5 ions per MS scan (with a dynamic exclusion of 10 s) with an AGC target of 2 × 10^5^ ions, a maximum ion injection time of 50 ms, and a resolution of 17,500.

### Metabolite identification and pathway analysis

Metabolomics raw data were pre-processed with Progenesis QI (Nonlinear Dynamics) in which peak picking, peak matching and annotation using between-subject experimental design were performed. Univariate analysis using one-way ANOVA followed by a post hoc comparison using a two-sided t-test to select differentially abundant mass ions between the two treatment groups was computed. The variances were not assumed to be equal between treatment groups, and the adjusted *p*-value cut-off threshold was set 0.05 following correction for multiple testing problem using FDR. The mass ions were identified using IDEOM with default parameters^45,46^ whereby retention time (RT) for the identification of authentic standards was set 5%, RT for the identification of calculated RT was set to 50%, and mass accuracy for mass identification was set to 5 ppm. Putative metabolites were also identified using the Human Metabolome Database (HMDB)^47^ and Progenesis MetaScope with a mass error threshold of 5 ppm.

Lipidomics raw datasets were processed using LipidSearch Software (Thermo Fisher Scientific). Lipid identification was based on the use of the Top 5 ddMS/MS, which uses an internal library and theoretical fragmentation with the addition of the ability to manually reject identifications that do not fit class trends in RT.

Metabolites and lipids were identified with four levels of confidence according to the classification of the Metabolomics Standards Initiative (MSI)^48–50^. In level 1 (L1) identification: the accurate masses, MS/MS fragmentation and retention times of the detected metabolite/lipid peaks were matched with those of the authentic standards which were co-analysed with the samples under identical experimental conditions. In level 2 (L2) identification, either the accurate masses and retention times of the detected metabolite peaks match with those of the authentic; or the accurate masses and MS/MS spectra were matched with compounds in a library when data was taken under the same acquisition parameters. In level 3 (L3) identification, predicted retention times or predicted MS/MS spectra or both were used due to the lack of standards. Finally, in level 4 (L4) identification, unambiguously assigned molecular formulas were used whereby insufficient evidence exists to propose possible structures.

The heatmap of the relative abundance levels of the identified metabolite was constructed using heatmapper^51^ and pathway analysis was performed using MetaboAnalyst 3.0^52^.

## Supplementary Information

Supplementary Information.

## Data Availability

The miRNA microarray data are available at NCBI's Gene Expression Omnibus (GEO) Series accession number GSEXXX (approval pending). The rest of the data that support the findings of this study are available on request from the corresponding author (H.A).
