# Supplementary material for: Intratumour heterogeneity in microRNAs expression regulates glioblastoma metabolism
Source: Sci Rep. 2021 Aug 5;11:15908. doi: 10.1038/s41598-021-95289-9 (PMC8342598; doi:10.1038/s41598-021-95289-9)
Supplement: Supplementary file 1 — Supplementary Information. [file 41598_2021_95289_MOESM1_ESM.docx]

**Supplementary Information: Intratumour Heterogeneity in MicroRNAs Expression Regulates Glioblastoma Metabolism**

Huda Alfardus^1,2^, Maria de los Angeles Estevez Cebrero^2^, Jonathan Rowlinson^2^, Anbarasu Lourdusamy^2^, Salah Abdelrazig^3^, Catherine Ortori^3^, Richard Grundy^2^, Dong-Hyun Kim^3^, Alan McIntyre^2^, Stuart Smith^2^

^2^School of Medicine, The University of Nottingham, Nottingham, NG7 2RD, United Kingdom

^3^Centre for Analytical Bioscience, Advanced Materials and Healthcare Technologies Division, School of Pharmacy, The University of Nottingham, UK.

^1^h.alfardus@leeds.ac.uk; huda.alfardus@nottingham.ac.uk

**Supplementary Table 1: Tumour sample information and clinicopathological characteristics of adult GBM cases used in the microarray.**

GBM patients from which tissue were collected were given numbers following the prefix GBM. Abbreviations: PCV: Procarbazine, CCNU, and Vincristine; BSC: best supportive care; RT/CT: radiotherapy/chemotherapy; N/A: not applicable; WT: wild-type; R132H: point mutation changing arginine at 132 into histidine; TMZ: temozolomide.

| **Tissue sample** | **Regions** | **Tumour site (lobe)** | **Resection status (%)** | **RT/CT** | **TMZ response** | **Overall survival**  **(months)** | **Age** | **Sex** | ***IDH*-1**  **status** | **ATRX**  **status** | **MGMT status** |
| --- | --- | --- | --- | --- | --- | --- | --- | --- | --- | --- | --- |
| **GBM 3.1** | rim | Right temporal | 100 | 60/TMZ | Good | 23.1 | 54 | F | WT | N/A | N/A |
| **GBM 3.2** | core |  |  |  |  |  |  |  |  |  |  |
| **GBM 3.4** | Invasive margin |  |  |  |  |  |  |  |  |  |  |
| **GBM 5.1** | rim | Left parietal, occipital | 100 | 60/TMZ | Moderate | Alive | 58 | F | WT | N/A | N/A |
| **GBM 5.2** | core |  |  |  |  |  |  |  |  |  |  |
| **GBM 5.5** | invasive margin |  |  |  |  |  |  |  |  |  |  |
| **GBM 9.1** | rim | Left frontal, | 100 | 60/TMZ |  | 17.2 | 48 | M | WT | N/A | N/A |
| **GBM 9.2** | Core |  |  |  |  |  |  |  |  |  |  |
| **GBM 9.5** | Invasive margin |  |  |  |  |  |  |  |  |  |  |
| **GBM 13.1** | rim | Right Parietal | 100 | 60/TMZ | Good | 9.7 | 26 | M | WT | N/A | N/A |
| **GBM 13.2** | core |  |  |  |  |  |  |  |  |  |  |
| **GBM 13.4** | invasive margin |  |  |  |  |  |  |  |  |  |  |
| **GBM 15.1** | rim | Right temporal | 100 | 60/TMZ | Good | 25.7 | 33 | F | R132H | N/A | Intermed |
| **GBM 15.3** | core |  |  |  |  |  |  |  |  |  |  |
| **GBM 15.5** | invasive margin |  |  |  |  |  |  |  |  |  |  |
| **GBM 17.1** | rim | Left frontal | 100 | 30 |  | 4.4 | 73 | F | WT | N/A | <10% |
| **GBM 17.2** | core |  |  |  |  |  |  |  |  |  |  |
| **GBM 17.4** | invasive margin |  |  |  |  |  |  |  |  |  |  |
| **GBM 20.1** | rim | Right frontal | 99 | 60/TMZ | Good | 15.3 | 71 | M | WT | N/A | N/A |
| **GBM 20.2** | core |  |  |  |  |  |  |  |  |  |  |
| **GBM 20.5** | invasive margin |  |  |  |  |  |  |  |  |  |  |
| **GBM 22.1** | rim | Left temporal | 95% |  |  | 5.4 | 35 | F | R132H | N/A | N/A |
| **GBM 22.3** | core |  |  |  |  |  |  |  |  |  |  |
| **GBM 22.5** | invasive margin |  |  |  |  |  |  |  |  |  |  |
| **GBM 23.1** | rim | Left parietal | 90% |  |  | 6.2 | 53 | M | WT | N/A | N/A |
| **GBM 23.2** | core |  |  |  |  |  |  |  |  |  |  |
| **GBM 23.5** | invasive margin |  |  |  |  |  |  |  |  |  |  |
| **GBM 25.1** | rim | Left parietal | 99% | 60/TMZ |  | 12.9 | 60 | F | WT | WT | 0% |
| **GBM 25.2** | core |  |  |  |  |  |  |  |  |  |  |
| **GBM 25.5** | invasive margin |  |  |  |  |  |  |  |  |  |  |
| **GBM 26.1** | rim | Left temporal | 100% | 60/TMZ |  | 6.8 | 56 | F | WT | WT | 0% |
| **GBM 26.2** | core |  |  |  |  |  |  |  |  |  |  |
| **GBM 26.6** | invasive margin |  |  |  |  |  |  |  |  |  |  |
| **GBM 27.1** | rim | Right temporal | 99% | 60/TMZ |  | Alive | 30 | F | WT | Mut | 75% |
| **GBM 27.2** | core |  |  |  |  |  |  |  |  |  |  |
| **GBM 27.5** | invasive margin |  |  |  |  |  |  |  |  |  |  |
| **GBM 28.1** | rim | Right frontal | 99% | BSC |  | 3 | 71 | M | WT | WT | 0% |
| **GBM 28.2** | core |  |  |  |  |  |  |  |  |  |  |
| **GBM 28.5** | invasive margin |  |  |  |  |  |  |  |  |  |  |
| **GBM 29.1** | rim | Left frontal | 100 |  |  | Alive | 67 | F | WT | WT | 0% |
| **GBM 29.3** | core |  |  |  |  |  |  |  |  |  |  |
| **GBM 29.5** | invasive margin |  |  |  |  |  |  |  |  |  |  |
| **GBM 30.1** | rim | Left frontal | 100 | BSC |  | 9.3 | 53 | M | WT | WT | 0% |
| **GBM 30.2** | core |  |  |  |  |  |  |  |  |  |  |
| **GBM 30.5** | invasive margin |  |  |  |  |  |  |  |  |  |  |
| **GBM 31.1** | rim | Right temporal | 100 | 60/TMZ |  | 16.1 | 57 | F | WT | WT | 0% |
| **GBM 31.2** | core |  |  |  |  |  |  |  |  |  |  |
| **GBM 31.5** | invasive margin |  |  |  |  |  |  |  |  |  |  |


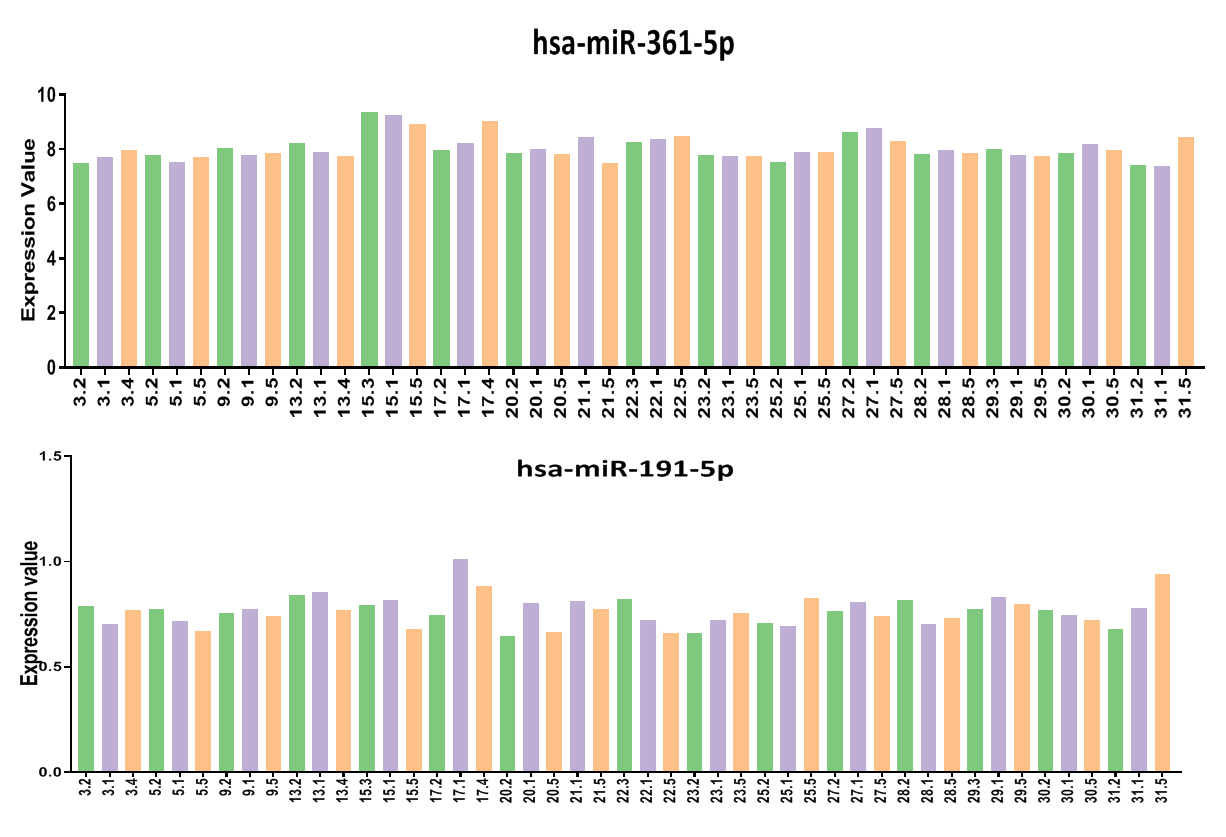


**Supplementary Figure 1: Microarray expression of housekeeping miRNAs.**

*Each bar represents one miRNA microarray expression value for (A) hsa-miR-361-5p and (B) hsa-miR-191-5p across tumour tissue samples (n=1). Green, purple and orange bars correspond to the core, rim and invasive margin tumour regions, respectively. Each bar is labelled with a patient number that corresponds to the number that comes after the prefix GBM in Supplementary Table 2.*


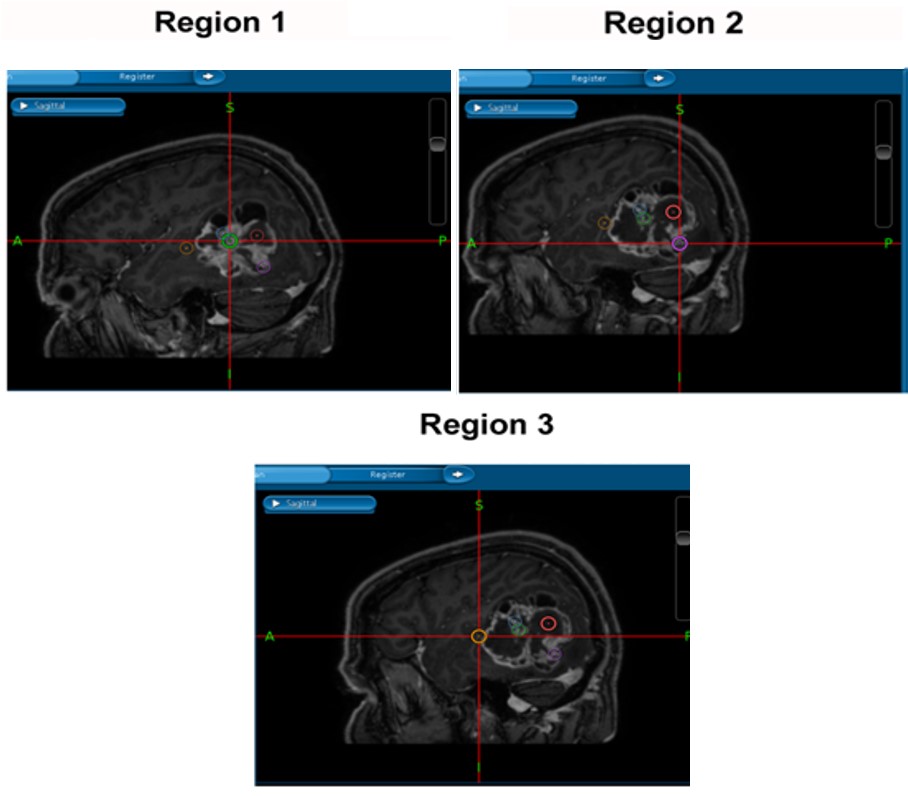


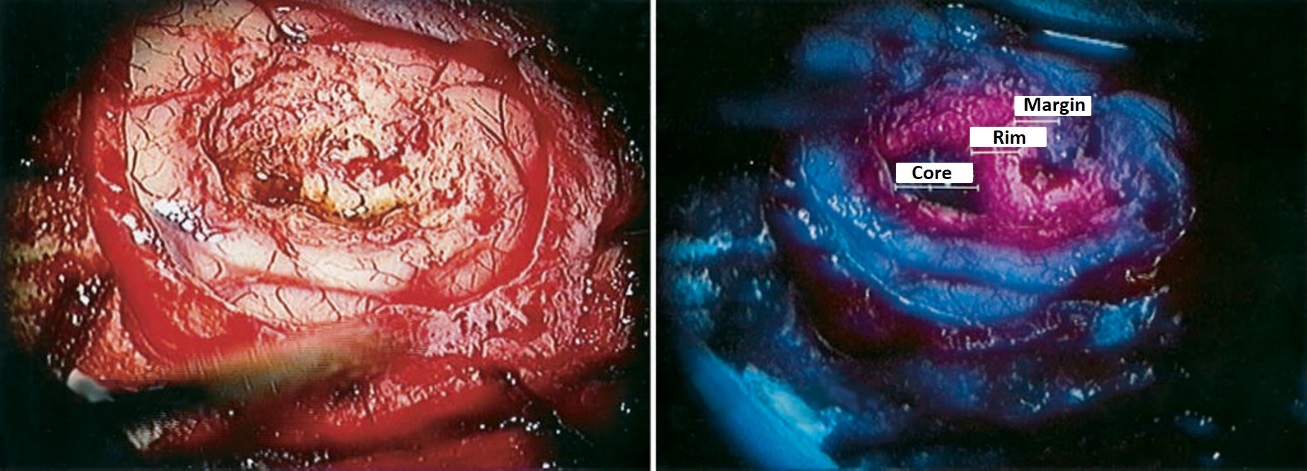


**Supplementary Figure 2:**

**Top image: Pre-operative MRI scans of a GBM patient’s brain allow the selection of different regions of the tumour.** *Regions 1, 2 and 3 represent the core, rim and invasive margin, respectively. Scans were captured using the StealthStation™ surgical navigation system (Medtronic, Dublin, Ireland).*

**Bottom image:** **Intra-operative images of a GBM patient’s tumour showing how 5-aminolevulinic acid (5-ALA) can aid the collection of tumour fragments from the core, proliferative rim and invasive margins of each tumour during surgery.** *The administration of 5-ALA allows the tumour to be visualised during the time of the surgery. This is because 5-ALA is converted into a fluorescent metabolite, protoporphyrin IX (PPIX) within tumour cells, but remains as a non-fluorescent compound* *in non-neoplastic cells. Left image: tumour viewed under conventional white light. Right image: tumour viewed under violet-blue illumination. Different regions can be selected based on the intensity of the fluorescence. Tumour core can be necrotic and appears non-florescent. The tumour rim is enhanced while the tumour margin (invasive regions) displays a low degree of fluorescence as the number of tumour cells is lower.*

*
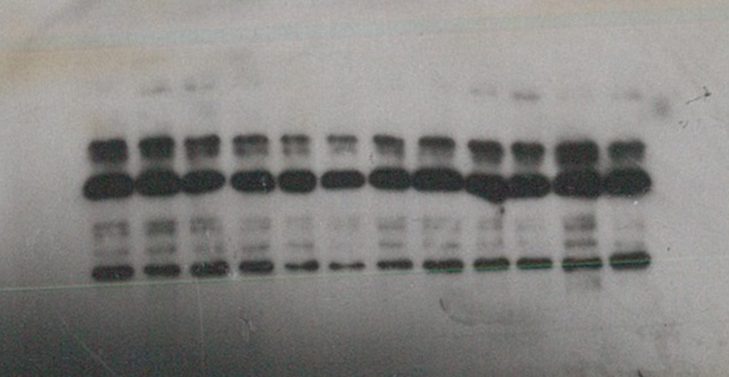
*

*
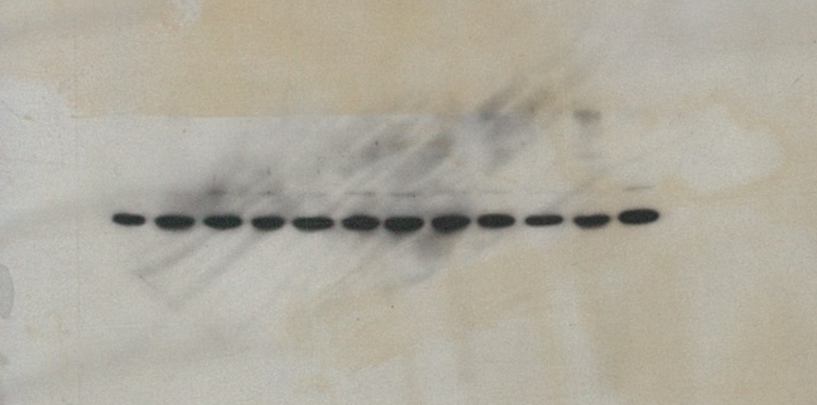
*

**Supplementary Figure 3:** *Full Western blot gel images. Top image: CPT2 immunostaining. Bottom image: GAPDH immunostaining.*
